# Gut Microbiota and Metabolic Alterations Associated with Heart Failure and Coronary Artery Disease

**DOI:** 10.3390/ijms252011295

**Published:** 2024-10-20

**Authors:** Adel A. Yafarova, Elena V. Dementeva, Olga A. Zlobovskaya, Anna F. Sheptulina, Elena V. Lopatukhina, Yuriy S. Timofeev, Evgeniya V. Glazunova, Aleksey V. Lyundup, Yuriy V. Doludin, Anton R. Kiselev, German A. Shipulin, Valentin V. Makarov, Oxana M. Drapkina, Sergey M. Yudin

**Affiliations:** 1 National Medical Research Center for Therapy and Preventive Medicine, Petroverigskyj Lane 10, Bld. 3, 101990 Moscow, Russiaantonkis@list.ru (A.R.K.);; 2 Federal State Budgetary Institution «Centre for Strategic Planning and Management of Biomedical Health Risks» of the Federal Medical and Biological Agency, Pogodinskaya Str., 10/1, 119121 Moscow, Russia; 3 Endocrinology Research Centre, Dmitry Ulyanov St. 19, 117036 Moscow, Russia

**Keywords:** gut microbiota, coronary artery disease, heart failure, dysbiosis, trimethylamine-N-oxide, alpha-diversity, beta-diversity, cardiovascular biomarkers

## Abstract

This study investigates the role of gut microbiota in cardiovascular diseases, with an additional focus on pro-atherogenic metabolites. We use advanced network analysis and machine learning techniques to identify key microbial features linked to coronary artery disease (CAD) and heart failure with reduced ejection fraction (HFrEF). This cross-sectional study included 189 participants divided into three groups: coronary artery disease (*n* = 93), heart failure with reduced ejection fraction (*n* = 43), and controls (*n* = 53). Assessments included physical exams, echocardiography, dietary surveys, blood analysis, and fecal analysis. Gut microbiota composition was analyzed using next-generation sequencing (NGS) and quantitative polymerase chain reaction (qPCR). Statistical analysis methods for testing hypotheses and correlations, alpha and beta-diversity analyses, co-occurrence networks, and machine learning were conducted using Python libraries or R packages with multiple comparisons corrected using the Benjamini–Hochberg procedure. Significant gut microbiota alterations were observed, with higher Bacillota/Bacteroidota ratios in CAD and HFrEF groups compared to controls (*p* < 0.001). Significant differences were observed in α-diversity indices (Pielou, Chao1, Faith) between disease groups and controls (*p* < 0.001). β-diversity analyses also revealed distinct microbial profiles (*p* = 0.0015). Interestingly, trimethylamine N-oxide (TMAO) levels were lower in CAD and HFrEF groups compared to controls (*p* < 0.05), while indoxyl sulfate (IS) levels were comparable between the study groups. Co-occurrence network analysis and machine learning identified key microbial features linked to these conditions, highlighting complex interactions within the gut microbiota associated with cardiovascular disease.

## 1. Introduction

The human microbiome consists of the collective genetic material of all the microorganisms—bacteria, fungi, protozoa, and viruses—that inhabit the human body, with the majority residing in the gut, particularly in the large intestine [1]. Over the past three decades, extensive research has significantly expanded understanding of gut microbiota (GM) beyond its traditional role in digestion, revealing its critical influence on cardiovascular health.

Gut microbiota (GM) and its particular metabolites have been implicated in the pathophysiology of atherosclerotic cardiovascular diseases (ASCVD) [2]. Circulating trimethylamine N-oxide (TMAO) and indoxyl sulfate (IS) are the most extensively studied proatherogenic metabolites of the gut microbiota. They are known to contribute to the pathogenesis of CVDs [2,3]. The majority of research has focused on the prognostic significance of elevated TMAO levels, particularly in atherosclerotic cardiovascular diseases (ASCVDs) [4,5,6,7,8]. Conversely, other TMAO precursors did not show this association [9]. In patients with heart failure with reduced ejection fraction (HFrEF), TMAO levels positively correlated with NYHA functional class and were higher than those in the control group [10]. Regarding indoxyl sulfate (IS), most studies on its role have focused exclusively on patients with the nephrology profile. However, recent evidence suggests that uremic toxins may also contribute to the increased incidence of ASCVD associated with chronic kidney disease (CKD) [11]. Despite the growing interest in the clinical significance of IS in CVDs, information on the relationship between serum IS and gut microbiota composition remains scarce.

Most studies have compared gut microbiota composition and its metabolites in patients with ASCVD to those in healthy controls. However, the temporal relationship between these changes and ASCVD development remains unclear. It is unknown whether altered gut microbiota precedes, results from, or simply co-occurs with ASCVD. A recent systematic review and meta-analysis of 21 studies revealed significant alterations in the gut microbiota associated with CAD. The analysis revealed changes in the abundance of four bacterial phyla: Alphaproteobacteria, Bacteroidota, Actinomycetota, and Bacillota. Notably, Gammaproteobacteria, *Enterobacteriaceae*, and *Escherichia* sp. showed increased abundance in CAD patients. Conversely, Bacteroidota, particularly the *Bacteroides* genus, exhibited a significant decline. Bacillota results were mixed, showing increases in *Lactobacillus* sp. and *Streptococcus* sp., while *Lachnospiraceae* showed a decrease. Additionally, a reduction was noted in the Coriobacteriales order of the Actinomycetota phylum. Less prominent changes were observed in *Desulfovibrio* sp., *Parabacteroides* sp., and *Fusobacterium* sp. [12]. A recent systematic review and meta-analysis revealed increased abundances of Alphaproteobacteria and Actinomycetota in the heart failure (HF) group, while Bacteroidota and the Bacillota/Bacteroidota ratio were lower. Although Bacillota showed a decreasing trend, it was not statistically significant. At the genus level, *Streptococcus* sp., *Bacteroides* sp., *Alistipes* sp., *Bifidobacterium* sp., *Escherichia-Shigella* sp., *Enterococcus* sp., and *Klebsiella* sp. showed increased abundance. Conversely, *Ruminococcus* sp., *Faecalibacterium* sp., *Dorea* sp., and *Megamonas* sp. showed reduced abundance. The analysis suggests that gut microbiota changes in CAD and HF are associated with alterations in bacterial abundance, disruptions in short-chain fatty acid (SCFA) production, and an increase in TMAO-producing bacteria [13].

This study aimed to comprehensively examine gut microbiota composition, including α- and β-diversity, and its associated pro-atherogenic metabolites (TMAO and IS) across different stages of the cardiovascular continuum. This research innovatively employs co-occurrence network analysis and machine learning techniques to explore microbial community connections and identify key gut microbiota features associated with CAD and HFrEF.

## 2. Results

### 2.1. Clinical Parameters

Clinical parameters between groups were evaluated with Mann–Whitney or χ^2^ (Fisher, if the expected frequencies in the contingency table were small) tests. We applied FDR correction for quantitative and qualitative characteristics, respectively.

Primary clinical features for all study groups are displayed in Supplementary Table S1(a,b). Patients in the disease groups were older than those in the comparison group. However, no statistically significant age differences were observed between the HFrEF and CAD groups (see Supplementary Table S1). BMI was comparable between groups (*p* > 0.05). The majority of patients with HFrEF (73.4%) were classified as NYHA class II, 13.3% as class I, and 13.3% as class III.

The HFrEF group exhibited statistically significantly lower GFR according to CKD-EPI (Chronic Kidney Disease Epidemiology Collaboration Formula) and creatinine clearance according to Cockcroft–Gault compared to the CAD and comparison groups. Transaminase activity and triglyceride concentrations were not significantly different between the studied groups. The LDL-C level was higher in the control group.

Supplementary Tables S2(a,b) present the main results of laboratory tests for each study group. Supplementary Tables S3(a,b) show statistically significant variances in the median values of echocardiographic parameters between groups.

Likewise, Supplementary Tables S4(a,b) present the main classes of medications. Statistical analysis revealed significant differences between study groups in medication use, except for antiarrhythmics.

The study groups showed no statistically significant differences in the frequency of consumption of individual food groups, except for red and white meat, fish, and simple carbohydrates, including starch. The scores according to the HEI-2020 index are presented in Supplementary Table S5.

**Figure 1:**
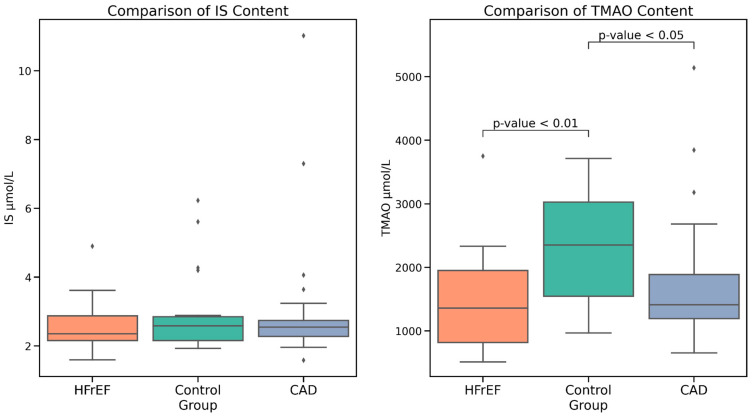
Serum IS and TMAO concentrations in patients with HFrEF, CAD, and the control group.

No statistically significant difference in serum IS levels was observed between the study groups (*p* = 0.378). Interestingly, both HFrEF and CAD groups exhibited statistically lower TMAO levels compared to the control group. Meanwhile, TMAO levels in the CAD group were similar to those in patients with HFrEF (Mann–Whitney test, Table 1, Figure 1). Notably, GLM with covariates (age, BMI, creatinine, and eEGFR) showed significance only for HFrEF and control groups (*p* = 0.002).

A positive correlation was observed between TMAO levels and fast carbohydrate intake in our study (Spearman r = 0.372, *p* = 0.011). The relative abundance of the Lachnospiraceae family also showed a positive correlation with TMAO levels (Spearman r = 0.372, *p* = 0.011). Interestingly, the Lachnospiraceae family is known to be a high-TMAO producer.

### 2.2. Microbiome Composition Assessment

#### α- and β-Diversity (Next-Generation Sequencing Data)

**Figure 2:**
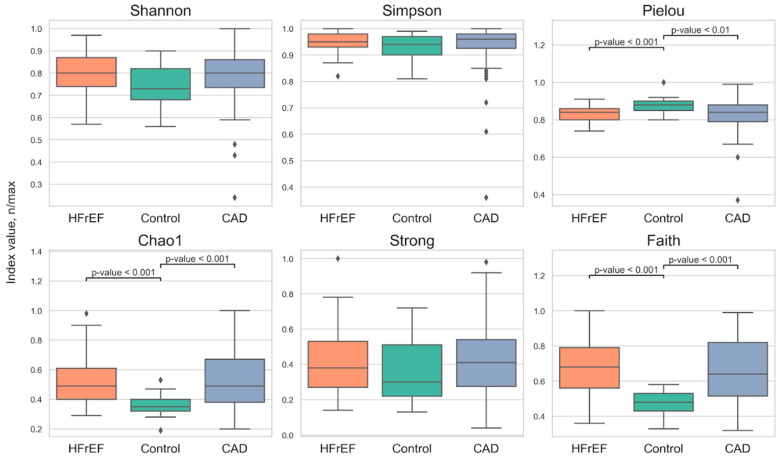
Alpha-diversity indices in the studied groups.

We estimated alpha-diversity using six indices: Shannon, Simpson, Pielou, Chao1, Strong, and Faith. Figure 2 and Table 2 (Mann–Whitney test *p*-values) show the results of alpha-diversity analysis (species diversity or richness within a functional community) across study groups. We conducted three tests: Kruskal–Wallis followed by the Dwass–Steel–Critchlow–Fligner test, GLM (quasi-Poisson, overdispersion), and Mann–Whitney. These tests produced similar results. Significant differences were observed in certain α-diversity indices (Pielou, Chao1, Faith) between both disease states and the control group. The Kruskal–Wallis test revealed *p*-values less than 0.001 for these indices across the three groups.

Shannon, Simpson, and Strong indices showed no significant differences between the groups. We also found no significant differences in α-diversity between HFrEF and CAD.

β-diversity quantifies the similarity or differences between microbial pairs, representing a fundamental characteristic of microbial communities [14]. Analysis of β-diversity allows us to associate patterns of taxonomic or functional diversity with environmental features, enabling predictions of ecosystem properties or the host’s health state. We assessed β-diversity using various models: Euclidean (Manhattan, Minkowski), Canberra, Bray–Curtis, and Jaccard. All these models showed statistically significant differences between each disease and control groups (*p* = 0.0015, PERMANOVA test, FDR corrected).

**Figure 3:**
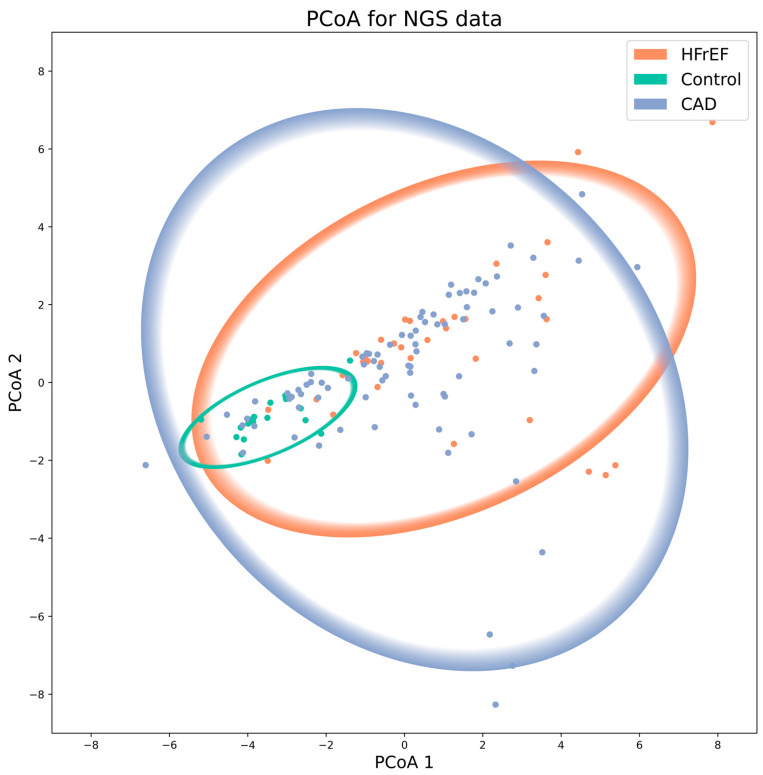
Principal Coordinates Analysis (PCoA) analysis of microbial communities in HFrEF, CAD, and control groups. Each point on the graph represents a single microbial community sample from the HFrEF, CAD, or control groups.

Figure 3 and Table 3 provide the β-diversity analysis of microbial communities using principal coordinates analysis (PCoA) with 95% confidence ellipses.

Since beta-diversity measures reflect differences in microbial community composition between samples, the data tends to be complex and multi-dimensional. The PCA method allows for reducing data dimensionality while preserving maximum variability. This analysis helps to pinpoint the specific microbial families contributing most to the observed differences, thus offering a deeper understanding of how ASCVDs influence gut microbiota composition. Microbial diversity analysis revealed significant separation between CAD, HFrEF cases, and controls after adjusting for age and BMI based on two principal components from PCA (Table 3, Mann–Whitney test *p*-values).

Eighteen microbial families contributed most to the differences in the PCA analysis (Table 4).

The assessment of α- and β-diversity indices revealed distinct microbial patterns in the CAD and HFrEF groups, with greater bacterial richness and phylogenetic diversity compared to the control group. Notably, no significant differences were observed between CAD and HFrEF groups, consistent with findings from β-diversity models.

### 2.3. Phyla Content Direct Comparison Between Groups

**Figure 4:**
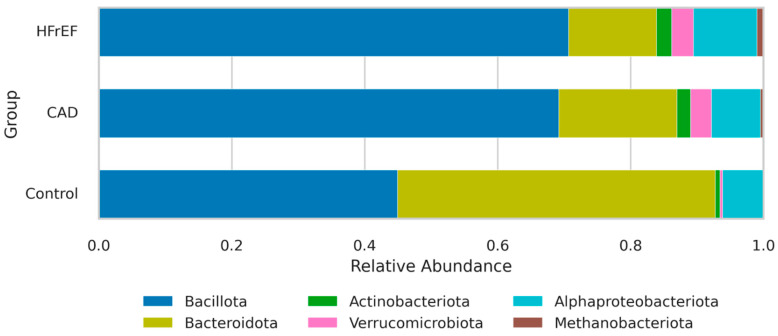
Histogram of the community composition of gut microbiota at the phylum level in patients with HFrEF, CAD, and the control group according to Next-Generation Sequencing data (RDP database).

Figure 4 illustrates the gut microbiota composition at the phylum level in patients with HFrEF, CAD, and the control group.

We assessed differences in the composition of CM between study groups using the method of generalized linear models (quasi-Poisson, overdispersion), controlling for age and BMI as covariates (see Supplementary Table S6).

This study found a significant increase in the abundance of Bacillota and Actinomycetota in both CAD and HFrEF groups compared to the control group (*p* < 0.001). Conversely, the Bacteroidota phylum showed significantly higher relative abundance in the control group compared to the CAD and HFrEF groups (*p* < 0.001). Accordingly, the Bacillota/Bacteroidota ratio was found to be significantly higher in the CAD and HFrEF groups compared to the control group.

The relative abundance of phyla was comparable in patients with CAD and HFrEF except for *Methanobacteriota,* which was higher in HFrEF.

### 2.4. Machine Learning Analysis

#### 2.4.1. Random Forest Parameters Optimization

Prior to conducting the random forest analysis, we optimized parameters to best distinguish between groups. The optimized parameters are shown in Supplementary Tables S7 and S8. We also compiled a balanced dataset in terms of the number of samples and age/BMI. This was necessary because otherwise, training would have been dominated by the CAD group (the largest sample).

#### 2.4.2. Training RF Model on Three Groups Altogether, CAD and Control, HFrEF and Control

The main features for each training model are provided in Supplementary Table S9 in descending order. When training the model to recognize all three groups, we found that the control was clearly separated from the disease groups. However, the disease groups themselves were often misclassified. When choosing other samples in the groups, the situation was reproduced each time: the control was well distinguished from the diseases, but CAD and HFrEF were not different from each other (see Table 5).

Machine learning using only the key families for CAD and control and HFrEF and control showed similar effectiveness to learning on all families.

For HFrEF and control both the original (17 control/37 HFrEF) and truncated (17 control/20 HFrEF) samples showed good differentiation. Notably, there were several unique main features for each sample. For original samples, these included *Acholeplasmataceae*, *Desulfobacteraceae*, *Oxalobacteraceae*, and *Selenomonadaceae.* In the case of truncated samples, *Bacillales Incertae Sedis XI*, *Pseudomonadaceae*, *Anaeroplasmataceae*, and *Gracilibacteraceae* had unique features compared to the truncated sample.

#### 2.4.3. CAD and HFrEF

Despite good results when training to differentiate the control group from each disease state, machine learning was unable to reliably differentiate between CAD and HFrEF even in the truncated samples, well balanced for age and BMI (see Table 6).

#### 2.4.4. Comparing the Results Between Models

When attempting to distinguish the control group and CAD, or the control group and HFrEF, most of the key families were shared, which may account for the poor discrimination between the two disease states. Unique features of CAD included *Enterobacteriaceae*, *Fusobacteriaceae*, *Gracilibacteraceae,* and *Peptoniphilaceae.* Conversely, *Akkermansiaceae*, *Chitinophagaceae*, *Methanobacteriaceae*, and tentatively, *Anaeroplasmataceae*, *Desulfobacteraceae*, *Pseudomonadaceae,* and *Selenomonadaceae* may be identified as unique features of HFrEF (see Table 7).

**Figure 5:**
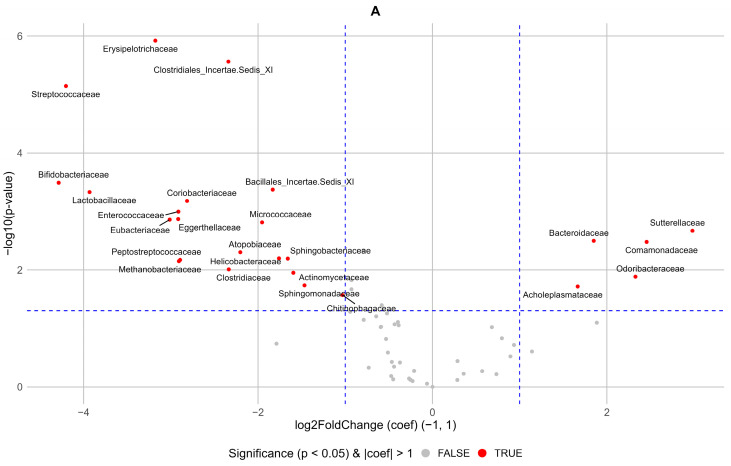

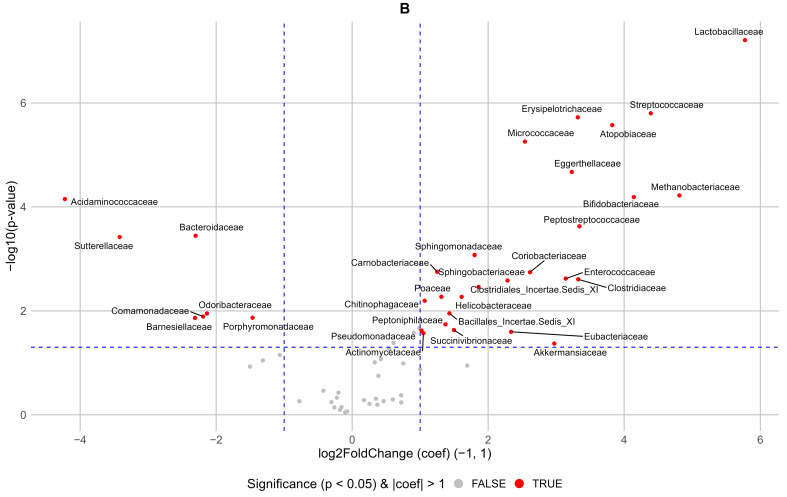
Volcano plots for the MaAsLin2 results. (**A**) CAD-control; (**B**) HFrEF-control.

We next analyzed whether the most important features identified by RF are also significant in the MaAsLin2 analysis (see Figure 5).

We next investigated whether the most important features identified by random forest were also significant in the MaAsLin2 analysis. Most features overlapped, particularly for the HFrEF-control pair, although both methods identified unique features. Supplementary Table S10 shows FDR *p*-values for the main random forest features in the MaAsLin2 analysis. MaAsLin2 analysis identified unique features, including *Actinomycetaceae* and *Methanobacteriaceae* for the CAD-control pair, and *Peptoniphilaceae* (*p* = 0.037), *Succinivibrionaceae* (*p* = 0.045), *Actinomycetaceae* (*p* = 0.047), and *Spirochaetaceae* (*p* = 0.047) for the HFrEF-control pair.

### 2.5. Co-Occurrence Networks

**Figure 6:**
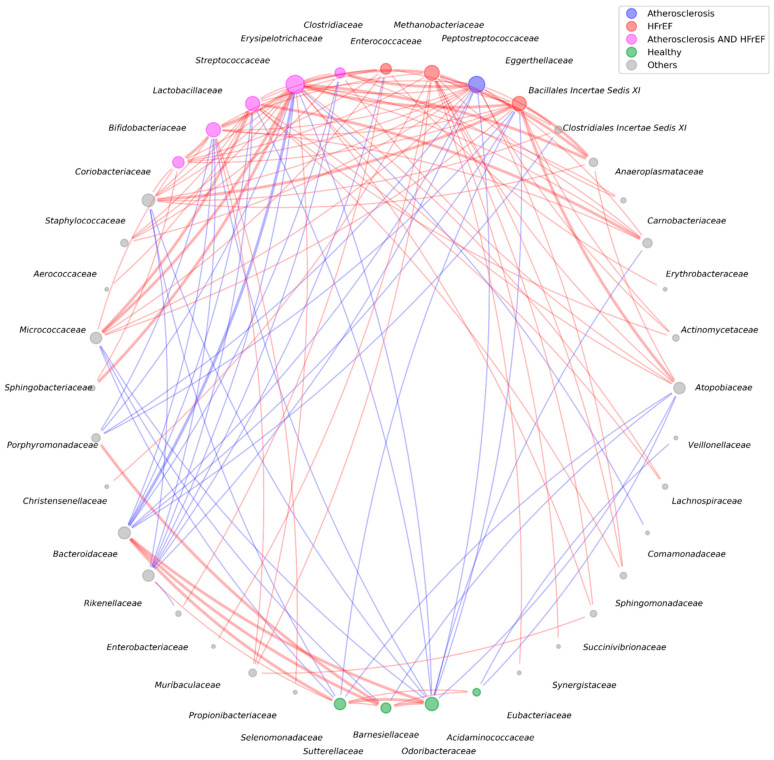
Microbial network for bacteria important for differentiating diseases and those with at least five nodes.

Co-occurrence networks can be used to explore interactions between different groups of microorganisms. Figure 6 and Supplementary Table S11 show the co-occurrence network, considering all key microorganisms from the three study groups.

Node colors represent microbial prevalence: red for HFrEF, blue for CAD, green for control, and purple for dominance in both disease groups compared to control. Gray nodes represent microorganisms not statistically prevalent in any group. Line color denotes a positive (red) or negative (blue) correlation between microorganisms. Bold lines (edges) refer to co-occurrences between microorganisms with moderate and strong correlations (Spearman coefficient of 0.5 and higher). Correlations are only shown for adjusted *p*-values less than 0.05.

## 3. Discussion

This study provides a comprehensive analysis of gut microbiota composition at the phylum level in patients with coronary artery disease (CAD) and heart failure with reduced ejection fraction (HFrEF) using next-generation sequencing (NGS) 16S and quantitative PCR (qPCR). We also assessed plasma concentrations of key gut microbiota metabolites, such as trimethylamine N-oxide (TMAO) and indoxyl sulfate, and explored microbial relationships through co-occurrence network analysis. Additionally, we applied machine learning techniques to identify taxa that differentiate CAD and HFrEF patients from the control group. Our findings partially align with existing literature, which documents distinct gut microbiota alterations across different stages of the cardiovascular continuum [12,15,16]. Notably, this investigation reveals novel associations between gut microbiota composition and clinical and instrumental parameters in patients with CAD and HFrEF.

### 3.1. Gut Microbiota Analysis

#### 3.1.1. F/B Ratio

The elevated Firmicutes-to-Bacteroidota (F/B) ratio observed in individuals with CAD suggests a similar elevation in patients with ischemic HFrEF, as confirmed by our study. This finding contrasts with the study by C.C.K. Mayerhofer et al. (2020), which reported a lower F/B ratio in HFrEF patients compared to healthy controls [17]. The discrepancy may stem from differences in study populations, as Mayerhofer’s study included patients with non-ischemic HFrEF, whereas our study focused solely on ischemic HFrEF. Our results are consistent with those of L. Cui et al. (2017) and T. Emoto et al. (2016), who found a higher relative abundance of Bacillota and reduced Bacteroidota in CAD patients compared to controls [18,19]. Variations in dietary conditions between study groups may account for discrepancies in findings across various studies. For example, W. Sun et al. (2021) reported a decrease in Bacteroidota and an increase in Actinomycetota in HFrEF patients, which we did not observe [16]. These differences may be due to the more severe symptoms in Sun’s study participants, classified predominantly as NYHA class III-IV, compared to our cohort, which mainly included NYHA class II patients.

#### 3.1.2. α- and β-Diversities

In assessing α- and β-diversities, our analysis revealed a greater number of unique taxonomic features in both CAD and HFrEF groups, aligning with the results of Z. Zhao et al. (2022) [20]. However, increased bacterial richness and phylogenetic diversity in these patients do not necessarily indicate a healthier gut microbiota. The observed elevation in Chao1 and Faith indices may reflect dysbiosis, characterized by an overabundance of opportunistic pathogens. The lower Pielou’s index in atherosclerotic cardiovascular disease (ASCVD) groups compared to controls further supports the presence of dysbiotic changes. Our results are consistent with L. Cui et al. (2017), who reported higher Shannon, Chao-1, and Ace indices in CAD patients [18]. However, α-diversity findings are mixed across studies, with some reporting no significant difference between HFrEF patients and controls [21,22], while others report reduced α-diversity in HFrEF [16,17]. Notably, patients with non-ischemic heart failure (*n* = 44) in Mayerhofer’s study exhibited significantly lower operational taxonomic units (OTUs) and Chao1 index values, likely reflecting the severity of their condition [17].

Our analysis of β-diversity demonstrated significant differences in microbial composition among CAD, HFrEF, and control groups, reinforcing the concept that gut dysbiosis is associated with cardiovascular disease. These findings align with prior research showing distinct microbial communities in CAD patients compared to healthy individuals, highlighting the importance of gut microbiota in cardiovascular health [23]. β-diversity differences across cardiovascular disease groups suggest that microbial diversity alterations could serve as potential markers for cardiovascular risk [24]. Collectively, these observations suggest that changes in α- and β-diversity in CAD patients reflect dysbiosis, characterized by an overabundance of pathogenic microorganisms rather than a diversity associated with a healthy gut microbiota.

#### 3.1.3. Correlation Networks

To better understand the interactions within the gut microbiota, we analyzed associations between bacterial families. These interactions may involve cross-feeding, quorum sensing, and functional redundancy among phylogenetically distinct microorganisms [25,26]. Our co-occurrence network analysis identified two distinct clusters of bacterial families: a disease state cluster and a control state cluster.

The control state cluster united the *Bacteroidaceae*, *Rikenellaceae*, *Sutterellaceae*, *Barnesiellaceae*, *Odoribacteraceae*, *Porphyromonadaceae* and *Acidaminococcaceae* families. These families are characteristic of a normal gut microbiota and are responsible for short-chain fatty acid production and amino acid fermentation.

We categorized families within the disease state cluster into several subgroups:

- Pathogenic or opportunistic families: *Streptococcaceae*, *Erysipelotrichaceae*, *Enterococcaceae*, *Peptostreptococcaceae*, *Actinomycetaceae*, *Atopobiaceae*, *Staphylococcaceae*, *Aerococcaceae*, *Micrococcaceae*, *Propionibacteriaceae*, *Sphingomonadaceae*, all of which may contribute to inflammation, especially in abundance.
- Neutral or beneficial families: *Methanobacteriaceae*, *Lactobacillaceae*, *Bifidobacteriaceae*, *Coriobacteriaceae*, and *Eggerthellaceae*, which could cause disease if present in abundance or if imbalanced with other beneficial microorganisms.
- Families with unascertained properties: *Anaeroplasmataceae*, *Carnobacteriaceae*, and *Erythrobacteraceae*, which require further investigation because their specific functions or metabolic roles have not been fully characterized or confirmed in the existing literature.

Opportunistic family *Atopobiaceae* from the disease cluster was inversely associated with unsaturated fat consumption (*p* < 0.001) in our study. Diets rich in unsaturated fats, such as those found in olive oil, nuts, and fish, are well known for their cardioprotective effects. Given that the *Atopobiaceae* family has been implicated in various inflammatory processes, its reduced presence in individuals with higher unsaturated fat consumption could have significant implications for cardiovascular health. The dietary intake of unsaturated fats may play a protective role in modulating gut microbiota by reducing the abundance of potentially harmful bacteria—or the lack of unsaturated fats in the diet may result in a better environment for these opportunistic bacteria.

Our study also identified a direct association between *Methanobacteriaceae* and *Christensenellaceae*, consistent with findings from the multi-ethnic HELIUS study [27]. This relationship may be explained by a cross-feeding mechanism, where hydrogen produced by *Christensenella minuta* serves as a substrate for *Methanobrevibacter smithii* [27]. We also observed correlations between the *Methanobacteriaceae*, *Atopobiaceae,* and *Veillonellaceae* families, with many genera in these families encoding the cutkC/D TMA-lyase enzyme, facilitating the synthesis of trimethylamine (TMA) from choline [28]. Archaea may then utilize TMA to produce methane [29].

We also observed positive correlations between *Lactobacillaceae*, *Clostridiaceae*, and *Bifidobacteriaceae* families. These families were also associated with the presence of either CAD or HFrEF. It is noteworthy that the genera *Clostridium*, *Lactobacillus*, and *Bifidobacterium* are capable of synthesizing secondary bile acids [30]. Previous studies have established that concentrations of secondary bile acids are elevated in patients with CAD and HFrEF compared to control groups [31,32,33].

Regarding *Erythrobacteraceae*, a less studied family from the disease cluster, we found various significant negative associations between the presence of this family and the following parameters: creatinine clearance (*p* = 0.007), eGFR (*p* = 0.024), and antiplatelet agent use (*p* = 0.02). *Erythrobacteraceae* abundance also showed a positive association with atrial fibrillation (*p* = 0.01). These findings suggest that *Erythrobacteraceae* may be involved in the complex interactions between gut microbiota, kidney function, and cardiovascular conditions. This relationship raises the possibility that *Erythrobacteraceae* may contribute to or serve as a marker of declining kidney health, which is closely associated with cardiovascular risk.

### 3.2. Gut Microbiota Metabolites: TMAO and Indoxyl Sulfate

Our study revealed significantly lower TMAO levels in CAD and HFrEF patients compared to controls, partially aligning with the findings of L. Bordoni et al. (2020), who reported lower TMA levels in CAD patients but comparable serum TMAO concentrations between groups [34]. Conversely, three recent studies reported significantly higher plasma TMAO levels in heart failure patients compared to controls [35,36,37], suggesting that HFrEF may alter gut microbiota composition and metabolic activity, leading to increased TMAO levels.

Several factors could explain the conflicting results of our study. Higher serum TMAO levels in controls may be due to greater red meat consumption, a primary source of TMAO precursors, compared to CAD and HFrEF patients. Differences in hepatic flavin monooxygenase activity, not assessed in our study, might also account for variability in serum TMAO concentrations. Additionally, medications like high-dose statins and acetylsalicylic acid, commonly used by CAD and HFpEF patients, have been shown to reduce serum TMAO levels significantly [38,39], potentially explaining the lower TMAO levels in our patient groups.

We found no significant differences in serum indoxyl sulfate (IS) concentrations between the study groups, which contrasts with other studies that have demonstrated direct associations between serum IS levels and cardiovascular parameters. For example, T.J. Lin et al. (2020) reported a correlation between serum IS and carotid-femoral pulse wave velocity in stage 2 CKD and CAD patients [40], while F.U. Dzgoeva et al. (2023) found associations between serum IS and both morphological and functional cardiac and aortic changes [41]. Elevated serum IS concentration was identified as an independent predictor of aortic stiffness in patients with CAD [40]. M. Imazu et al. (2017) first demonstrated that plasma IS levels were higher in heart failure patients compared to controls with similar GFR, suggesting that heart failure may independently increase plasma IS levels, irrespective of kidney function. Imazu et al. (2017) also found that plasma IS levels were closely associated with left ventricular systolic dysfunction in HF patients. Furthermore, a decrease in plasma IS concentration, achieved using a specific adsorbent, had a beneficial effect on the course of HF [42].

The absence of severe kidney dysfunction in our cohort may partially explain the discrepancy between our findings and those of previous studies.

### 3.3. Machine Learning

We applied the random forest (RF) machine learning method to assess the potential of differentiating disease groups from controls based on gut microbiota composition. Using leave-one-out cross-validation, the model successfully distinguished CAD and HFrEF patients from controls. However, it could not differentiate between CAD and HFrEF due to the high similarity of their metagenomes. Our study demonstrates the potential of machine learning in identifying microbial markers for cardiovascular diseases, though it is not yet ready for clinical application.

The RF model identified several families as critical for differentiating disease groups from controls, with considerable overlap between the CAD and HFrEF groups. These families include *Streptococcaceae*, *Ruminococcaceae*, *Erysipelotrichaceae*, *Clostridiales Incertae Sedis XI*, *Atopobiaceae*, *Bacteroidaceae*, *Coriobacteriaceae*, *Bifidobacteriaceae*, *Eggerthellaceae*, *Odoribacteraceae*, *Eubacteriaceae*, *Acidaminococcaceae*, *Micrococcaceae*, *Sphingomonadaceae*, *Rikenellaceae*, *Desulfovibrionaceae*, *Peptostreptococcaceae*, *Porphyromonadaceae*, *Clostridiaceae*, *Comamonadaceae*, *Christensenellaceae*, *Oxalobacteraceae*, *Enterococcaceae*, *Prevotellaceae*, *Barnesiellaceae*, *Muribaculaceae*, *Lachnospiraceae*, *Veillonellaceae*, and *Acholeplasmataceae*.

The following families were uniquely characteristic when differentiating CAD from the control group: *Enterobacteriaceae*, *Fusobacteriaceae*, *Gracilibacteraceae*, and *Peptoniphilaceae*. It is noteworthy that most genera within the *Enterobacteriaceae* family possess the enzyme choline TMA lyase and are capable of synthesizing TMAO from various substrates [35]. Additionally, members of this family can produce uremic toxins, contributing to the aggressive progression of atherosclerosis [43]. Jia-Lu Hu et al. (2021) found that a higher abundance of *Fusobacteriaceae* was associated with a reduced risk of CAD [44]. However, an earlier study linked members of this family to the development of atherosclerosis [45]. The association between *Gracilibacteraceae* and *Peptoniphilaceae* families and CAD has not been yet reported. Finally, the *Desulfovibrionaceae* family was positively correlated with salt intake in our study (*p* = 0.001).

The following families are uniquely characteristic when differentiating HFrEF from the control group: Akkermansiaceae, Chitinophagaceae, Methanobacteriaceae, Anaeroplasmataceae, Desulfobacteraceae, Pseudomonadaceae, and Selenomonadaceae. Akkermansia muciniphila, the most well-known member of the Akkermansiaceae family, is known to have cardioprotective properties [46,47]. However, data on its relative abundance in patients with HFrEF are lacking. In turn, it is known that the Chitinophagaceae family is an abundant family in the gut microbiota in patients with aortic valve calcification [48]. The Anaeroplasmataceae, Desulfobacteraceae, Pseudomonadaceae, and Selenomonadaceae families have not previously been associated with HFrEF. However, M. Jin et al. (2019) published data suggesting a role for the genus Anaeroplasma in preventing atherosclerosis [49].

*Methanobrevibacter smithii*, a key member of the *Methanobacteriaceae* family, is the most abundant methanogen in the gut microbiota. M.V. Fadeeva et al. (2020) demonstrated a higher relative abundance of *Methanobacteriaceae* in patients with HFrEF [50]. Previous research observed an excess of methanogens in the gut microbiota of individuals with obesity [51]. Most methanogens, such as *Methanobrevibacter smithii*, and members of the *Methanomassiliicoccus* genus, perform anaerobic respiration via the hydrogenotrophic pathway, utilizing hydrogen to produce methane from CO_2_ [52,53]. This pathway of methanogenesis needs hydrogen to produce methane, thus promoting fermentation and bacterial overgrowth. This leads to hydrogen accumulation, inhibiting gut bacteria metabolism and NAD+ regeneration from NADH [54,55]. Furthermore, we previously demonstrated a correlation between *Methanobacteriaceae* abundance and the history of ventricular tachycardia episodes in patients with HFrEF [56].

The top five families most significant for differentiating patients with HFrEF from controls included *Streptococcaceae*, *Erysipelotrichaceae*, *Ruminococcaceae*, *Acidaminococcaceae*, and *Atopobiaceae.* A recently published systematic review on gut microbiota composition in HFrEF patients indirectly confirms the findings regarding the *Streptococcaceae* family. In addition, several other studies have reported increased relative abundance of the *Streptococcus* genus in HFrEF [57,58]. Moreover, a large study involving 8973 asymptomatic CAD patients from the SCAPIS (Swedish CArdioPulmonary bioImage Study) highlights the association between specific *Streptococcus* genus and CVDs [59]. Species such as *S. parasanguinis*, *S. gordonii*, and *S. agalactiae* were found to be positively associated with coronary artery calcium scores. Comparing participants with high and low abundance of these coronary artery calcium score-associated species (using a cutoff median), those with a higher abundance of *S. anginosus* and *S. oralis* subsp. *oralis* generally exhibited more cardiovascular risk factors. It is noteworthy that participants in this study had not received prior medication for CAD, allowing for an assessment of gut microbiota composition and its association with the coronary calcium index and several other parameters without drug influence. Similar associations were observed in previously published case-control studies [60,61,62]. Furthermore, abundance of specific *Streptococcus* species in the gut microbiota of patients with CVDs was linked to elevated high-sensitivity C-reactive protein levels in the blood, as well as leukocytosis and neutrophilia [59].

Data on the prevalence of the *Erysipelotrichaceae* family, which carries TMA lyases [63] and produces SCFAs [64], in patients with CVDs, are limited. For example, Z. Jie et al. (2017) found an increased abundance of *Erysipelotrichaceae* to be associated with atherosclerosis and coronary artery disease [60]. In contrast, another study found a lower abundance of *Erysipelotrichaceae* in patients with HFrEF compared to healthy controls. This discrepancy may be due to the inclusion of patients with HFpEF of both ischemic and non-ischemic etiologies, with 70% of patients in the latter study being in a decompensated state [65]. Interestingly, *Erysipelotrichaceae* abundance was significantly higher in patients with large-artery atherosclerosis stroke compared to asymptomatic controls [64].

The *Acidaminococcaceae* family was significantly more abundant in the control group compared to the CAD and HFpEF groups. Information about the metabolism and physiology of the *Acidaminococcaceae* family is currently limited. Limited studies have indicated that members of the *Acidaminococcaceae* family can synthesize SCFAs, particularly butyrate [66,67]. One more study examining the gut microbiota in patients with CAD showed that the *Acidaminococcaceae* family was dominant in the control group [19]. B. Qi et al. (2023) found that the *Acidaminococcaceae* family played a protective role in patients with ischemic cardiomyopathy [68].

Most features identified as important by the RF model were found to be differentially abundant by MaAsLin2 analysis, although not all features overlapped. Several interpretations and suggestions can help explain this discrepancy. The RF model may have identified features that are critical in combination with other features, even if these features do not show significant individual differences in abundance. The RF model can also capture non-linear relationships. In contrast, MaAsLin2 may have missed these features if they lacked a strong univariate signal. Furthermore, features essential for RF classification may not be statistically significant in MaAsLin2 due to differences in sensitivity or significance threshold stringency. The RF model may be more sensitive to features contributing to overall classification, even if those features do not show strong differential abundance due to variability or subtle signal changes. Features identified by RF but not MaAsLin2 may represent complex, context-dependent associations with the disease that are not solely captured by abundance changes. They may be involved in pathways, networks (see the Section 3.1.3 Correlation Networks), or interactions crucial in the disease process but not necessarily reflected solely by their abundance.

Little information was found in the literature on machine learning-based gut microbiome analysis in patients with CVDs. To date, only one study has been published on controlled machine learning based on gut microbiota composition analysis for CVD screening [69]. In a study titled The American Gut Project, samples were collected from 478 patients with CVD and 473 individuals without CVD. Significant differences were observed between the CVD and non-CVD groups at the level of 39 taxa. Specifically, the CVD group exhibited higher abundances of *Bacteroides*, *Subdoligranulum*, *Clostridium*, *Megasphaera*, *Eubacterium*, *Veillonella*, *Acidaminococcus*, and *Listeria* genera. Conversely, the non-CVD group showed higher abundances of *Faecalibacterium*, *Ruminococcus*, *Proteus*, *Lachnospira*, *Brevundimonas*, *Alistipes*, and *Neisseria* genera. Machine learning algorithms trained on the 39 distinctive taxa achieved a maximum AUC of approximately 0.58 (using RF and neural networks). Subsequently, machine learning models were trained using the 500 OTUs with the highest variance, increasing the AUC to approximately 0.65 (using random forest). Further refining the selection to the top 25 taxonomic features increased the AUC to approximately 0.70, which is considered satisfactory. However, achieving very high AUC values is challenging due to the high variability and complex interplay of multiple factors influencing microbiota data. Limitations of The American Gut Project study included the lack of data on sex, age, and BMI, as well as the broad categorization of patients into CVD and non-CVD groups. CVD encompasses a wide range of pathologies, making it inappropriate to group all these heterogeneous conditions together. Furthermore, the publication lacks information on medication therapy received by patients.

Our findings suggest that while machine learning can differentiate between cardiovascular disease and control groups, it remains challenging to distinguish between specific cardiovascular conditions due to metagenomic similarities. The model identified specific bacterial families that significantly contributed to the classification, suggesting potential microbial markers for cardiovascular diseases.

### 3.4. Limitations

This study has several limitations. First, the relatively small sample size may have limited the statistical power of the findings, especially in the context of machine learning analyses. Second, the cross-sectional design of the study limits the ability to establish causality between gut microbiota and cardiovascular diseases. Longitudinal studies would be essential to confirm whether the observed microbiota alterations are causes or consequences of CAD and HFrEF. Third, although we did not observe significant differences in dietary habits among participants, this is likely due to the homogeneity of our cohort. The participants were predominantly individuals residing in the same geographical region and sharing similar dietary patterns. Such uniformity may have limited our ability to detect substantial diet-related variations in gut microbiota composition. Additionally, dietary data relied on self-reported questionnaires, potentially introducing bias due to the subjective nature of responses. Furthermore, we did not account for all potential confounding factors, such as medication use and lifestyle habits, which may have influenced gut microbiota composition and related metabolic profiles. Finally, the analysis focused on specific microbial metabolites, and other potentially relevant metabolites were not evaluated. This may have overlooked important aspects of gut microbiota interactions with cardiovascular health.

## 4. Materials and Methods

**Figure 7:**
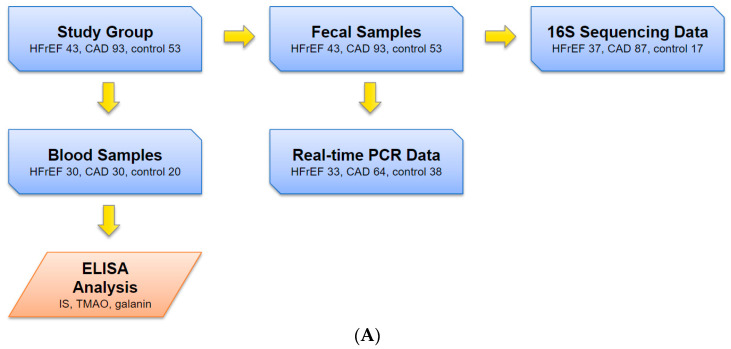

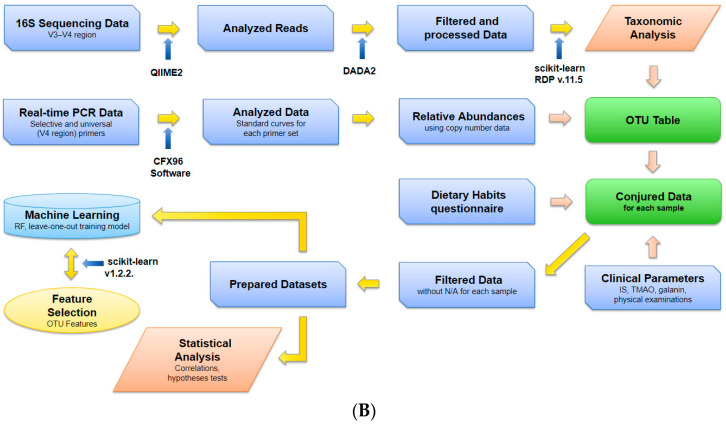
Study design: (**A**) samples and data collection; (**B**) data analysis. HFrEF—Heart Failure with reduced Ejection Fraction; CAD—Coronary Artery Disease; IS—Indoxyl Sulfate; TMAO—trimethylamine N-oxide; QIIME2—Quantitative Insights Into Microbial Ecology; DADA2—Divisive Amplicon Denoising Algorithm; RDP—Ribosomal Database Project; OTU—Operational Taxonomic Unit; N/A—Not Available; RF—Random Forest.

The overall process is described in Figure 7A,B.

Initial data collection involved analyzing the medical histories and outpatient records of patients either hospitalized or monitored at the outpatient clinic of the National Medical Research Center for Therapy and Preventive Medicine of the Ministry of Health of Russia. The following clinical characteristics of the study participants were analyzed: age, sex, body mass index (BMI), NYHA functional class in patients with HFrEF; history of myocardial infarction, myocardial revascularization (percutaneous coronary intervention/coronary artery bypass grafting), catheter ablation, implantation of cardiac resynchronization therapy with a defibrillator, arterial hypertension, presence of atrial fibrillation/flutter, and episodes of ventricular tachycardia in the medical history. The medication therapy taken by the patients was also analyzed. Potential candidates meeting inclusion criteria individually received detailed information about the research project, agreed to participate, and provided informed consent for participation, data processing, and biobanking of blood and stool samples. Blood samples were collected from all patients in a fasting state for standard clinical blood tests (complete blood count and biochemical blood analysis) and for biobanking, as well as stool samples for biobanking and subsequent microbiome analysis using various methods. Creatinine clearance was calculated for all patients using the Cockcroft–Gault formula (measured in mL/min), and glomerular filtration rate (GFR) was calculated using the CKD-EPI formula (measured in mL/min/1.73 m^2^). Additionally, all patients underwent transthoracic echocardiography with Doppler assessment of transmitral flow and tissue Doppler imaging. Dietary assessment was conducted using the Healthy Eating Index-2020 questionnaire.

At the second stage, patient samples were selected based on inclusion and exclusion criteria. Of the 250 patients initially recruited, 135 were included in the metagenomic analysis: 43 with CAD and HFrEF, 93 with CAD but no HFrEF, and 53 without cardiovascular disease (control group). We first analyzed gut microbiome composition using biobanked biomaterials via next-generation sequencing (after all quality checks producing 37 HFrEF, 87 CAD, 17 control results). The remaining extracted DNA was used for the real-time PCR (33 HFrEF, 64 CAD, 38 control results).

At the third stage, a subgroup of 80 male patients was selected from the total sample (*n* = 135), comprising 30 with CAD and HFrEF, 30 with CAD without HFrEF, and 20 individuals without cardiovascular disease. These patients underwent analysis of their gut microbiome metabolites (TMAO and IS).

### 4.1. Participants

The study involved three groups of unique participants: those with HFrEF (*n* = 43), those with CAD (*n* = 93), and a comparison group (*n* = 53).

Inclusion criteria for the HFrEF group included: signs and symptoms of heart failure, left ventricular ejection fraction (LVEF) ≤ 40%, and confirmed ischemic etiology. Ischemic etiology was defined as previous percutaneous coronary intervention (PCI), coronary artery bypass grafting (CABG), or myocardial infarction (MI).

Inclusion criteria for the CAD group included: verified stable coronary artery disease (previous PCI, CABG, or MI) with no heart failure symptoms or signs. An additional exclusion criterion for the CAD group was a score greater than 5 on the HFA-PEFF scale to rule out patients with HFpEF.

Inclusion criteria for the control group included: the absence of CVD and other established diseases based on medical history and records.

Exclusion criteria for all groups included: refusal to participate, BMI ≥ 35 kg/m^2^, GFR < 30 mL/min/1.73 m^2^ (CKD-EPI formula), smoking within the last 10 years, and a history of chronic obstructive pulmonary disease (COPD) or asthma (moderate to severe). Other exclusion criteria included: systemic connective tissue diseases, untreated oncological diseases, recent acute or exacerbated chronic infections, pregnancy or lactation, inflammatory bowel diseases, and recent use of antimicrobials, probiotics, systemic glucocorticosteroids, immunosuppressants, or laxatives within the last 3 months.

Assessments included physical examinations, electrocardiography, B- and M-mode echocardiography, dietary habit evaluation using questionnaires, and collection of blood and fecal samples for analysis and biobanking as we described previously [70]. Next, metabolites (trimethylamine N-oxide, indoxyl sulfate) were analyzed in a subgroup of 80 male patients: 30 with HFrEF, 30 patients with CAD, and 20 controls. The research adhered to the principles outlined in the Declaration of Helsinki and its subsequent revisions. The study was approved by the Institutional Ethics Committee of the National Medical Research Center for Therapy and Preventive Medicine, Moscow, Russia (protocol No. 04-05/18, dated 7 June 2018, and protocol No. 03-02/19, dated 11 April 2019). Before data collection commenced, participants were provided with a comprehensive project explanation; they signed informed consent and were enrolled in the study between the years 2018 and 2020.

### 4.2. Procedure for Biobanking Serum and Plasma Samples

Certified nurses collected venous blood samples in the procedure room only after patients signed informed consent for biobanking. Blood collection was performed using a vacuum tube with a separating gel for serum collection and a vacuum tube with K2EDTA for plasma collection. Biological material processing and storage were conducted in the biobank of the National Medical Research Center for Therapy and Preventive Medicine, Ministry of Health of Russia.. We prepared samples using standard procedures, including centrifugation and subsequent aliquoting. Serum and plasma were obtained by centrifuging primary vacuum tubes containing patient biomaterial within 30 min of blood collection. Centrifugation was performed for 15 min at 4 °C and 1200 rcf (2800 rpm) using a Centrifuge 5702 R. After centrifugation, the tubes were visually inspected for hemolysis and lipemia. The samples were then transferred into labeled cryotubes in volumes of 500 and 1000 µL using an automatic pipette with disposable filter tips (100–1000 µL). All collected samples were stored in a freezer at –72 °C. Biobank samples were stored in a depersonalized form under a unique patient ID, adhering to ethical standards. Each cryotube containing biological material was linked to information about the biosample (type, volume, storage location, etc.) and the patient ID in the biobank’s information system via a unique barcode. Before analysis, samples were thawed at room temperature.

### 4.3. Procedure for Stool Sample Collection by the Patient

All participants received a sterile plastic container with a lid and toilet insert for stool collection, along with instructions for collection and storage. Participants refrained from intense physical activity, alcohol consumption, and dietary changes for 24 h prior to stool collection. The sample was transported in a cooler bag with cold packs to the laboratory at the Centre for Strategic Planning and Management of Biomedical Health Risks of the Federal Medical Biological Agency within no more than 2 h.

### 4.4. Evaluation of Indoxyl Sulfate and Trimethylamine-N-Oxide

The evaluation of biomarker levels involved measuring indoxyl sulfate (IS) and trimethylamine N-oxide (TMAO) in blood serum after a period of overnight fasting. These samples were processed in accordance with BRISQ guidelines and stored at temperatures ranging from −70 °C to −80 °C. 

IS quantification was performed using a human indoxyl sulfate (IS) ELISA kit (Blue Gene Biotech, Shanghai, China), with a measurement range of 1–25 µmol/L and an analytical sensitivity of 0.1 µmol/L. We used an ELISA kit for trimethylamine oxide (Cloud-Clone Corp, Wuhan, China/Katy, TX, USA) to quantify TMAO, with a measurement range from 123.5 to 10,000.0 pg/mL and an analytical sensitivity of 54.1 pg/mL.

### 4.5. Dietary Habits

Dietary habits were evaluated using the adapted Healthy Eating Index 2020 questionnaire (HEI-2020) [71], which assessed the frequency of consuming specific food groups. The HEI-2020 questionnaire was designed to assess dietary alignment with recommendations for 2020–2025. The questionnaire consists of 14 questions that assess the frequency of consuming specific food groups. Each question is scored from 0 to 3 points (e.g., fresh fruits and vegetables, meat, fish, and salt) or from 0 to 10 points (e.g., dietary fiber, animal and plant protein, added sugars, and dairy products). Scores are assigned based on adherence to recommended minimum and maximum consumption levels.

### 4.6. Fecal Sample Analysis

DNA was extracted using QIAamp kits (either QIAamp^®^ Fast DNA Stool Mini or QIAamp^®^ PowerFecal Kit; Qiagen, Hilden, Germany), and concentrations were measured with a Qubit fluorometer (dsDNA HS Assay Kit; Thermo Fisher Scientific, Waltham, MA, USA). PCR amplification using gene-specific primers targeted the V3–V4 regions of 16S rRNA. DNA libraries were prepared, quality-checked with a Bioanalyzer, and then sequenced on the Illumina MiSeq platform. Reads were processed in QIIME2, including trimming, filtering, and merging via DADA2 to generate amplicon sequence variants (ASVs). Taxonomy assignment was performed using a Naive-Bayes classifier trained on SILVA v.138.99 and RDP v.11.5 reference 16S rRNA databases. Rarefaction curves indicated an appropriate 19 k sampling depth for analysis. Samples were as follows: HFrEF (*n* = 37), CAD (*n* = 87), and a comparison group (*n* = 17).

We also used quantitative polymerase chain reaction (qPCR) to assess the abundance of several taxa, including *Enterobacteriaceae*, *Lactobacillaceae*, and *Christensenellaceae*; genera *Bacteroides* sp., *Bifidobacterium* sp., *Odoribacter* sp., Oscillibacter sp., *Ruminococcus* sp., and *Subdoligranulum* sp.; species *Enterococcus faecalis*, *Faecalibacterium prausnitzii*, and total bacterial load. We used standard curves for each primer set to determine DNA concentration based on cycle threshold (Ct) values. The formula used was C = 10^(Ct−int)/slope^, where C is the initial target concentration in gene copies per μL, Ct is the cycle threshold for the sample, and “int” represents the intercept. We calculated OTU or total bacterial concentrations using corresponding NCBI data, accounting for 263 varying 16S rRNA gene copy numbers in bacterial species. OTU were then normalized to the total bacterial concentration in each sample to calculate relative abundance. Samples were as follows: HFrEF (*n* = 33), CAD (*n* = 64), and a comparison group (*n* = 38).

A detailed description of the experiments can be found in our previous article [70].

### 4.7. Statistical Analysis

Statistical analyses were primarily conducted in Python 3.8. We used Pandas v1.5.3 and NumPy v1.23.5 for general tasks such as dataset preparation. For statistical processing, we utilized SciPy v1.10.1 and statsmodels v0.14.1. These libraries were used for:

- Shapiro–Wilk test for normality checks
- Spearman test for correlations

For hypothesis testing:

- Mann–Whitney test
- Kruskal–Wallis test followed by Dwass–Steel–Critchlow–Fligner pairwise comparisons
- Generalized linear model (quasi-Poisson model type)
- χ^2^ test (or Fisher’s exact test if expected frequencies in the contingency table were small).

Beta-diversity (Bray–Curtis dissimilarity, Jaccard, Canberra distance, Euclidean distance, Manhattan distance, Minkowski distance) was evaluated using PERMANOVA (skbio.stats.distance from scikit-bio v0.5.9).

Principal coordinate analysis (PCoA), principal component analysis (PCA), and machine learning were performed using scikit-learn version 1.2.2. The machine learning approach utilized random forest (RF) with leave-one-out cross-validation (LOOCV).

We used seaborn v0.12.2 and matplotlib v3.7.1 to create graphs for the analyses. Network graphs were constructed using the NetworkX 3.1 package for Python 3.11.9.

Alpha-diversity was assessed using the R package vegan version 2.6-4 (R version 4.3.1) for the Shannon, Simpson, Pielou, Chao1, and Strong indices. Boxplots were generated using ggplot2 version 3.4.2. The indices were compared using the R package jmv. The Faith index was calculated in two steps: constructing a taxonomic tree using species names (https://timetree.org/, accessed on 10 June 2024), followed by index evaluation using the Python skbio.diversity library.

To correct for multiple comparisons in all comparison tests, we applied the Benjamini–Hochberg procedure, using the false discovery rate (FDR) correction implemented in the statsmodels.stats.multitest package.

MaAsLin2 (Multivariate Association with Linear Models) analysis was conducted with R package (MaAsLin2 from Bioconductor) with TSS parameter for normalization, LOG transformation of data, and Benjamini–Hochberg correction. The volcano plot was built with ggplot2 version 3.4.2.

## 5. Conclusions

Cardiovascular diseases remain the leading cause of chronic non-communicable diseases, significantly contributing to global morbidity and mortality rates. A novel mechanism of CVD pathogenesis referred to as the “gut–heart axis” is increasingly recognized by recently published literature. This emerging concept highlights the interaction between the cardiovascular system and the gut in the development and progression of CVDs. Our study supports the notion that the pathogenesis of atherosclerosis and related cardiovascular diseases is driven by consortia of microorganisms rather than individual species. We demonstrated, for the first time, that the composition of gut microbiota and its metabolic activity in individuals with stable CAD and ischemic HFrEF largely overlap. These findings underscore the importance of considering the complex interactions within the gut microbiota when investigating the microbial contributions to cardiovascular health. Further research is needed to refine these insights and explore their potential clinical applications.

## Figures and Tables

**Table 1:** Serum IS and TMAO concentrations in patients with HFrEF, CAD, and the control group.

Marker	HFrEF (*n* = 30)	CAD (*n* = 30)	Control Group (*n* = 20)	*p*
IS, µmol/L	2.36 [2.16, 2.88]	2.55 [2.28, 2.74]	2.59 [2.16, 2.85]	>0.05
TMAO, µmol/L	1361 [818, 1951]	1413 [1196, 1889]	2352 [1547, 3028]	*p*_1-2_: >0.05
				*p*_2-3_: 0.047
				*p*_1-3_: 0.007

HFrEF—Heart Failure with reduced Ejection Fraction; CAD—Coronary Artery Disease; IS—Indoxyl Sulfate; TMAO—trimethylamine N-oxide.

**Table 2:** Alpha-diversity indices in the studied groups.

α-Diversity Indices	CAD	HFrEF	Control	HFrEF-CAD, *p*	HFrEF-Control, *p*	CAD-Control, *p*
Shannon	0.8 [0.74, 0.86]	0.8 [0.74, 0.87]	0.73 [0.68, 0.82]	>0.05		
Simpson	0.96 [0.93, 0.98]	0.95 [0.93, 0.98]	0.94 [0.9, 0.97]	>0.05		
Pielou	0.84 [0.79, 0.88]	0.84 [0.8, 0.86]	0.88 [0.86, 0.9]	>0.05	< 0.001	0.004
Chao1	0.49 [0.38, 0.67]	0.49 [0.4, 0.61]	0.35 [0.32, 0.4]	>0.05	< 0.001	< 0.001
Strong	0.41 [0.27, 0.54]	0.38 [0.27, 0.53]	0.3 [0.22, 0.51]	>0.05		
Faith	0.64 [0.52, 0.82]	0.68 [0.56, 0.79]	0.48 [0.43, 0.53]	>0.05	< 0.001	< 0.001

**Table 3:** Comparison of principal components between groups.

Components	HFrEF-Control Group, *p*	CAD-Control Group, *p*	HfrEF-CAD, *p*
Principal component 1	<0.001	<0.001	>0.05
Principal component 2	<0.001	0.003	>0.05

**Table 4:** The contribution of certain microbial families to differences in PCA analysis.

Microbial Families	Principal Component 1	Principal Component 2
*Actinomycetaceae*	0.130	−0.152
*Anaeroplasmataceae*	0.186	0.205
*Bacteroidaceae*	−0.178	0.100
*Barnesiellaceae*	−0.174	0.073
*Carnobacteriaceae*	0.153	−0.114
*Coriobacteriaceae*	0.144	−0.142
*Eggerthellaceae*	0.128	−0.129
*Erysipelotrichaceae*	0.230	0.023
*Fibrobacteraceae*	0.133	0.148
*Helicobacteraceae*	0.201	0.290
*Odoribacteraceae*	−0.189	0.067
*Pirellulaceae*	0.111	0.140
*Porphyromonadaceae*	−0.174	0.101
*Propionibacteriaceae*	0.183	0.263
*Rikenellaceae*	−0.212	0.099
*Sphaerochaetaceae*	0.139	0.241
*Sphingomonadaceae*	0.202	0.015
*Streptococcaceae*	0.154	−0.127

**Table 5:** Random Forest results when training on different combinations of groups.

Groups	Features Selected	CAD	Control	HFrEF
		*n* Correct/*n* Total (Percent)		
CAD-Control-HFrEF	no	9/20 (45%)	14/17 (82.4%)	10/20 (50%)
	no	8/20 (40%)	15/17 (88.2%)	12/20 (60%)
CAD-Control	no	16/20 (80%)	15/17 (88.2%)	-
	yes	16/20 (80%)	15/17 (88.2%)	-
Control-HFrEF (original samples)	no	-	15/17 (88.2%)	34/37 (91.9%)
	yes	-	16/17 (94.1%)	34/37 (91.9%)
Control-HFrEF (truncated samples)	no	-	16/17 (94.1%)	19/20 (95%)
	yes	-	17/17 (100%)	18/20 (90%)

**Table 6:** Random Forest results when training on CAD and HFrEF groups.

Group	Mean Age, y	Mean BMI	Correct Prediction, n/Total (%)
CAD	68.0	29.2	32/44 (72.7%)
HFrEF	68.4	29.0	8/36 (21.6%)

**Table 7:** Comparison of significant features among different learning models.

Family	CAD + HFrEF + Control	CAD + Control	HFrEF + Control	
	Trunc *	Trunc	Trunc	Orig *
*Acholeplasmataceae*		✔		✔
*Acidaminococcaceae*	✔	✔	✔	✔
*Akkermansiaceae*	✔		✔	✔
*Anaeroplasmataceae*			✔	
*Atopobiaceae*	✔	✔	✔	✔
*Bacillales Incertae Sedis XI*		✔	✔	
*Bacteroidaceae*	✔	✔	✔	✔
*Barnesiellaceae*	✔	✔	✔	✔
*Bifidobacteriaceae*	✔	✔	✔	✔
*Carnobacteriaceae*		✔	✔	✔
*Chitinophagaceae*			✔	✔
*Christensenellaceae*		✔	✔	✔
*Clostridiaceae*	✔	✔	✔	✔
*Clostridiales Incertae Sedis XI*	✔	✔	✔	✔
*Comamonadaceae*		✔	✔	✔
*Coriobacteriaceae*	✔	✔	✔	✔
*Desulfobacteraceae*				✔
*Desulfovibrionaceae*	✔	✔	✔	✔
*Eggerthellaceae*	✔	✔	✔	✔
*Enterobacteriaceae*	✔	✔		
*Enterococcaceae*	✔	✔	✔	✔
*Erysipelotrichaceae*	✔	✔	✔	✔
*Eubacteriaceae*	✔	✔	✔	✔
*Fusobacteriaceae*		✔		
*Gracilibacteraceae*		✔		
*Helicobacteraceae*		✔	✔	
*Lachnospiraceae*	✔	✔	✔	✔
*Lactobacillaceae*	✔	✔	✔	✔
*Methanobacteriaceae*	✔		✔	✔
*Micrococcaceae*	✔	✔	✔	✔
*Muribaculaceae*		✔	✔	✔
*Odoribacteraceae*	✔	✔	✔	✔
*Oxalobacteraceae*	✔	✔		✔
*Peptoniphilaceae*		✔		
*Peptostreptococcaceae*	✔	✔	✔	✔
*Porphyromonadaceae*	✔	✔	✔	✔
*Prevotellaceae*	✔	✔	✔	✔
*Pseudomonadaceae*			✔	
*Rikenellaceae*	✔	✔	✔	✔
*Ruminococcaceae*	✔	✔	✔	✔
*Selenomonadaceae*				✔
*Sphingobacteriaceae*		✔	✔	✔
*Sphingomonadaceae*		✔	✔	✔
*Streptococcaceae*	✔	✔	✔	✔
*Sutterellaceae*	✔	✔	✔	✔
*Veillonellaceae*	✔	✔	✔	✔

* trunc = truncated samples, orig = original samples. Red filling indicates the families present only in full or truncated HFrEF sample; orange filling marks the key features of only one disease state.

## Data Availability

The datasets used and analyzed in the present study are available from the NCBI database (Accession: PRJNA1165666, SRA submission SUB14744196).

## Supplementary Materials

The following supporting information can be downloaded at: https://www.mdpi.com/article/10.3390/ijms252011295/s1.
